# Changes in the ornithine cycle following ionising radiation cause a cytotoxic conditioning of the culture medium of H35 hepatoma cells

**DOI:** 10.1038/sj.bjc.6600700

**Published:** 2003-02-10

**Authors:** J van Rijn, J van den Berg, T Teerlink, F A E Kruyt, D S M Schor, A C Renardel de Lavalette, T K van den Berg, C Jakobs, B J Slotman

**Affiliations:** 1 Radiation Oncology, VU University Medical Center, PO Box 7057, 1007 MB Amsterdam, The Netherlands; 2 Metabolic Unit, Department of Clinical Chemistry, VU University Medical Center, PO Box 7057, 1007 MB Amsterdam, The Netherlands; 3 Medical Oncology, VU University Medical Center, PO Box 7057, 1007 MB Amsterdam, The Netherlands; 4 Molecular Cell Biology, VU University Medical Center, PO Box 7057, 1007 MB Amsterdam, The Netherlands

**Keywords:** X-rays, cytotoxic medium conditioning, ornithine cycle, hyperammonia

## Abstract

Cultured H35 hepatoma cells release a cytotoxic factor in response to irradiation with X-rays. When the conditioned medium from irradiated cells is given to nonirradiated cells, growth is inhibited and followed by cell death, possibly apoptosis, Analysis of the conditioned medium reveals a dramatic change in the ornithine (urea) cycle components after the irradiation. A strong decrease in medium arginine is accompanied with parallel increases in ornithine, citrulline and ammonia. The high level of ammonia appears to be largely responsible for the observed cytotoxicity. The development of hyperammonia by irradiated cells and the related toxicity depend on the radiation dose and the number of cells seeded thereafter for the medium conditioning. Development of cytotoxicity by irradiated cells is completely prevented with the arginase inhibitor L-norvaline, in arginine-deficient medium or when citrulline replaces arginine. These preventive measures result in subtoxic ammonia levels.

The classic test for the effect of ionising radiation on cell survival is the clonogenic assay, which determines the clonogenic capacity of individual cells (Puck and Markus, 1956). To obtain a reliable number of colonies when the expected survival is lower, more cells need to be seeded per flask. This may influence the assay and lead to distorted outcomes, as we discovered in radiation studies with the Reuber H35 cell line. When subsequent to irradiation with X-rays, cells are seeded at densities of ⩾400 cm^−2^, colonies become smaller and finally at 2000 cells cm^−2^ there is no colony formation at all. Only a change of the culture medium at the appropriate moment allows the surviving clonogenic cells to develop into colonies. When the conditioned medium is transferred to nonirradiated cells, it also precludes their colony formation. It was thus concluded that cytotoxic conditioning of the culture medium by the irradiated cells prevents (clonogenic) cell growth.

During the last decade several studies appeared about cytotoxic ‘bystander’ effects in irradiated cell cultures (reviewed by Iyer and Lehnert, 2000). Cytotoxic and clastogenic effects were detected on nonirradiated cells in contact with irradiated cells or exchanged via the culture medium (Hallahan *et al*, 1989; Mothersill and Seymour, 1997; Bishayee *et al*, 2001; Lewis *et al*, 2001; Sawant *et al*, 2002). The contribution of such bystander effects to radiation-induced cell death can be significant. When in human fibroblast cultures a single cell was targeted with helium ions, 80–100 damaged cells were detected on distant locations (Belyakov *et al*, 2001). Radiation-induced bystander effects are probably not restricted to stationary *in vitro* conditions as they were also observed *in vivo* in a mixed transplant of irradiated and nonirradiated bone marrow cells (Watson *et al*, 2000). Radiation-induced bystander effects are probably multifactorial and many of the described phenomena appear to be cell-type-specific.

The present study investigates a novel kind of cytotoxic conditioning in the culture medium of H35 hepatoma cells in response to treatment with X-rays.

## MATERIALS AND METHODS

H35 hepatoma cells, subclone KRC-7, were originally isolated from a minimal deviation hepatoma of the rat (Reuber, 1961). They have retained many of the metabolic characteristics of the hepatocytes from which they probably descend (Pitot *et al*, 1964). The cells were cultured in 25 cm^2^ tissue culture flasks with 5 ml L15 medium supplied with 2 mM glutamine in addition (final concentration 2.5–3 mM) and 10% foetal bovine serum. The experiments started with 72 h exponentially growing cultures with approximately 1.25×10^6^ cells. The medium was renewed 24 h before the treatments. Irradiation was done with 80 kV X-rays at a dose rate of 1 Gy min^−1^ (Pantak Therapax SXT 150). Then the cells were trypsinised and the suspension was used for the clonogenic assay or for the initiation of conditioning experiments with various cell densities. Irradiation and preparation of the cell suspensions took place at room temperature and were completed in less than 15 min. Nonirradiated cultures were manipulated in the same way (sham-irradiated). For the clonogenic assay, colonies were fixed after 8 days and stained with Giemsa solution. Conditioned media from experimental cultures were collected at different times and passed through a 22 *μ*m filter in order to test their growth-promoting capacities or to perform the various analyses. Data are the mean of duplicate tests. Standard errors were less than 5% and are therefore omitted. Experiments were repeated to ascertain good reproducibility. For the analysis of DNA, cells were collected by trypsinisation, centrifuged, washed and stored at −20°C in ethanol until further preparation. The method that categorises and quantifies DNA with the fluorescent propidium iodide was performed as described before (Kruyt *et al*, 1996). Amino acids including ornithine and citrulline were detected by high-performance liquid chromatography. The method is based on automated precolumn derivatisation of amino acids with orthophthalaldehyde, separation of the derivatives by reversed-phase chromatography and quantification by fluorescence detection (Teerlink *et al*, 1994). Ammonia was determined with a kit based on the reductive amination of 2-oxoglutarate using glutamate dehydrogenase and NADPH. The oxidation of NADPH was measured at 340 nm in a spectrophotometer and is proportional to the ammonia concentration. Chemicals and ammonia kit were obtained from Sigma Chemicals (St Louis, USA). Cell culture media including arginine-deficient Leibovitch L15 medium were purchased by GIBCO Life Technologies (Alphen a/d Rijn, The Netherlands).

## RESULTS

### Effect of conditioned medium from irradiated cells on cell proliferation and survival

When survival following a treatment is expected to be lower, more cells have to be seeded in the clonogenic assay. When H35 cells are treated with higher doses of X-rays, this results in smaller colonies or none at all. For example, when after treatment with 8 Gy X-rays H35 cells with a plating efficiency of 90–100% are seeded at a density of 5000 per flask, 30 clones are formed on average, which means that the surviving fraction is about 0.6%. However, when 50 000 cells are seeded, of the expected 300 colonies none are perceptable. Microscopic examination of developing microcolonies revealed a deterioration during the second half of the assay period of 8 days before the colonies attain the minimum size of 50 cells, which is acquired to count as a surviving cell. The expected 10-fold number of colonies in this example with 50 000 cells only shows up when the medium is changed at day 4 of the assay period. An earlier or delayed medium change results in a partial restoration of the colony formation or in smaller colonies. No restoration occurs when the medium is changed too early, that is, within 3 days following irradiation (results not shown).

**Figure 1:**
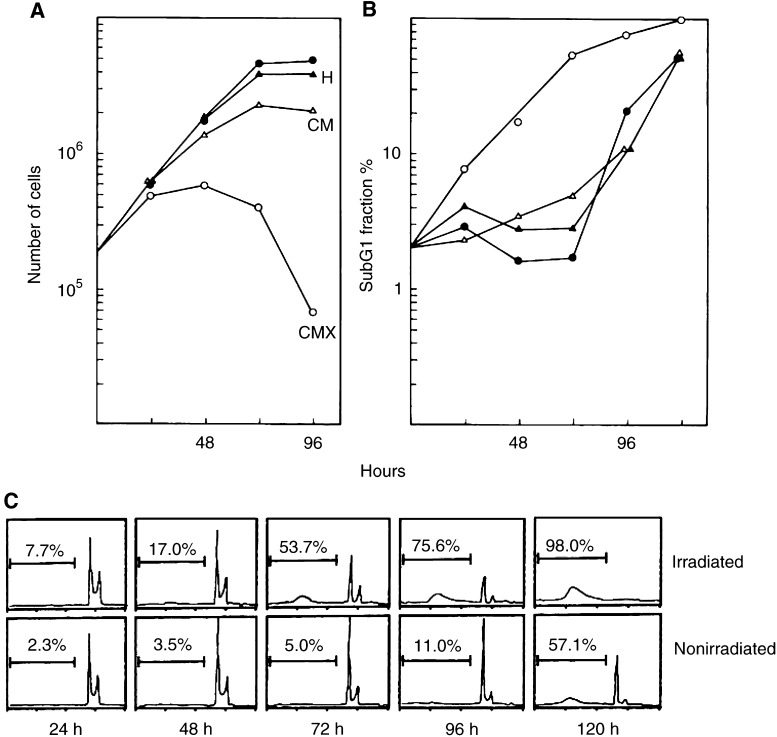
Cell proliferation in conditioned medium from irradiated cells and analysis of DNA. For a determination of the growth-promoting capacity, conditioned media from 96 h cell cultures were added to 24 h test cultures inoculated with 50 000 cells. Test cells were counted daily in combination with a clonogenic assay. (**A**). Parallel cultures were prepared for DNA analysis and the sub-G1 fraction (**B**) was determined from the DNA histograms (**C**). Cells were incubated in fresh medium (closed circles), or in conditioned medium from irradiated cells (CMX), that is, after irradiation with 8 Gy, 50 000 cells were seeded from which approximately 65 000 were present at 96 h. Alternatively, cells were grown in conditioned medium from 50 000 nonirradiated cells (CM) with a final density at 96 h of 2.5×10^6^. For a comparison, cells were also incubated at 42.5°C for 90 min (H), an isotoxic treatment that kills approximately 95% of the cells (Van Rijn *et al*, 2000). At variance with X-rays, hyperthermic cell death follows within a few hours and a couple of thousand cells were present in cultures with the surviving cells after the 96 h conditioning period. Except for 72 and 96 h with CMX, the clonogenic capacity of the test cell cultures was unaffected (data not shown).

It thus became clear that culture medium with irradiated cells acquires cytotoxic properties, which prevents surviving clonogenic cells from developing into colonies. The cytotoxic effect is further demonstrated when conditioned medium from irradiated cells (CMX) is given to nonirradiated cells, which then also prevents their colony formation. The effect of CMX on the proliferation of nonirradiated cells is shown in Figure 1

. Exponentially growing test cultures were changed to 96 h conditioned media from different origins, and cell growth and survival were monitored. In CMX, cell proliferation stops within 48 h, which is followed by a regression (Figure 1a). The growth inhibition is accompanied with a lower clonogenic capacity of the remaining viable cells, whereas significant numbers of cells become detached and do not form colonies in the assay (data not shown). In contrast to CMX, conditioned medium from nonirradiated cells (CM) causes no loss of the clonogenic capacity and much less inhibition of cell growth. The difference is striking since in untreated cultures the cell number increases from 50 000 to about 2.5×10^6^ during the 96 h of conditioning, whereas after 8 Gy only 60 000–80 000 cells are counted.

Using phase contrast time-lapse cinematography, we observed increasing numbers of blebbing cells after a few days. This indicates the possibility of apoptosis. Therefore, DNA was prepared from the cells and analysed with flow cytometry (Figure 1c). The emergence of a sub-G1 fraction in the DNA histograms along with the incubation in CMX is distinctive for the presence of fragmented DNA, which further indicates the possibility of apoptotic cell death. The time-dependent appearance of sub-G1 DNA in the presence of CMX is nearly exponential and starts immediately after the addition of CMX (Figure 1b). With fresh medium or conditioned media from untreated or heated cells, no increase in the sub-G1 fraction is seen until after 72 h, when the test cell population reaches its stationary growth phase.

**Figure 2:**
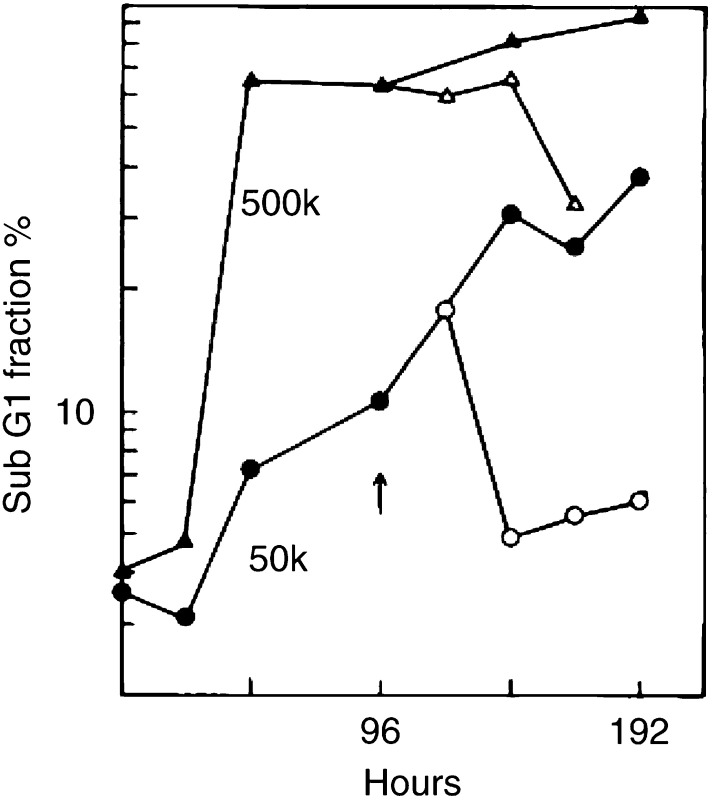
Sub-G1 DNA fractions in irradiated cell cultures with different initial cell numbers. After irradiation with 8 Gy X-rays, 50 000 (50 k) or 500 000 (500 k) cells were seeded. DNA analysis was performed in accordance with the time span used for the clonogenic assay. Cultures were either left undisturbed (closed symbols) or received fresh medium after 4 days (open symbols).

It was expected that CMX-induced DNA fragmentation is also detectable in the irradiated cell cultures. Figure 2

shows the formation of a sub-G1 DNA fraction after 8 Gy X-rays in cultures with 50 000 (50 k) or 500 k cells. At the highest density, the percentage sub-G1 DNA increases more than 10-fold during the second day already. With 50 k cells the increase is slower and proceeds within 6 days. When at day 4 the medium is changed in the low-density cultures, the sub-G1 fraction decreases about five-fold a few days later. In the high-density cultures no such recovery occurs.

### Effect of cell density and radiation dose on toxicity of CMX

**Figure 3:**
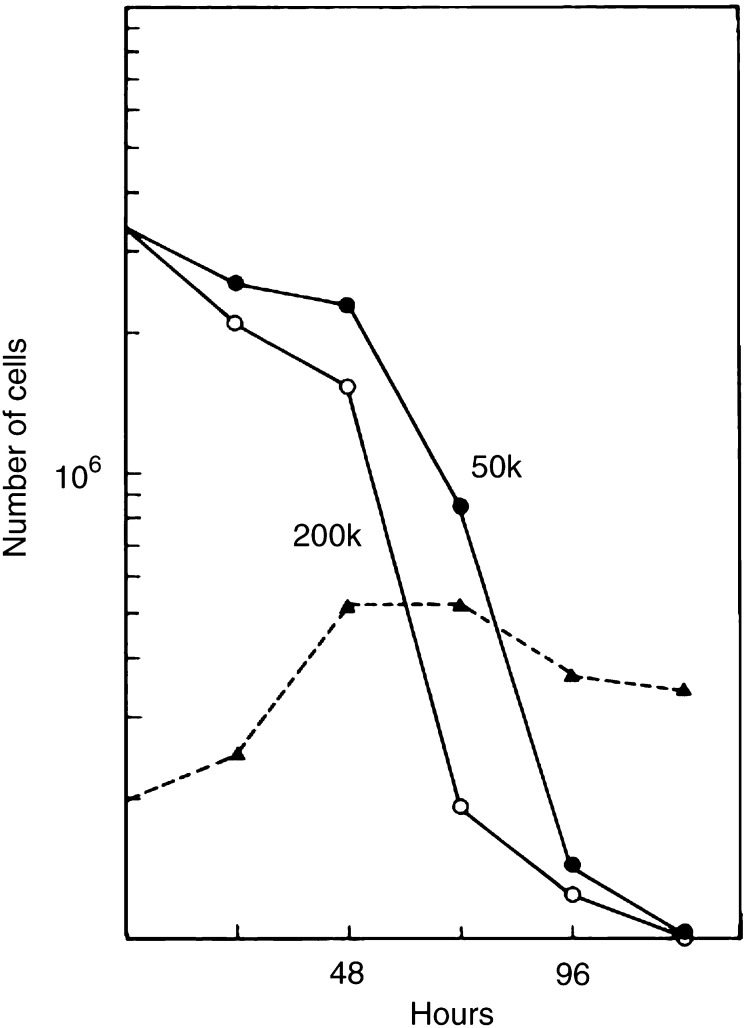
Effect of the cell density on the conditioning of the medium by irradiated cells. Irradiated cells (8 Gy) were seeded at densities 50 000 (50 k) and 200 000 (200 k) and the growth-promoting capacity of the CMX was tested at various times thereafter. Test cells were counted after 72 h in CMX. Cloning efficiencies were used to calculate the number of surviving cells as shown. The dashed line shows the number of cells (surviving and nonsurviving) that were present in the 200 k donor cultures.

**Figure 4:**
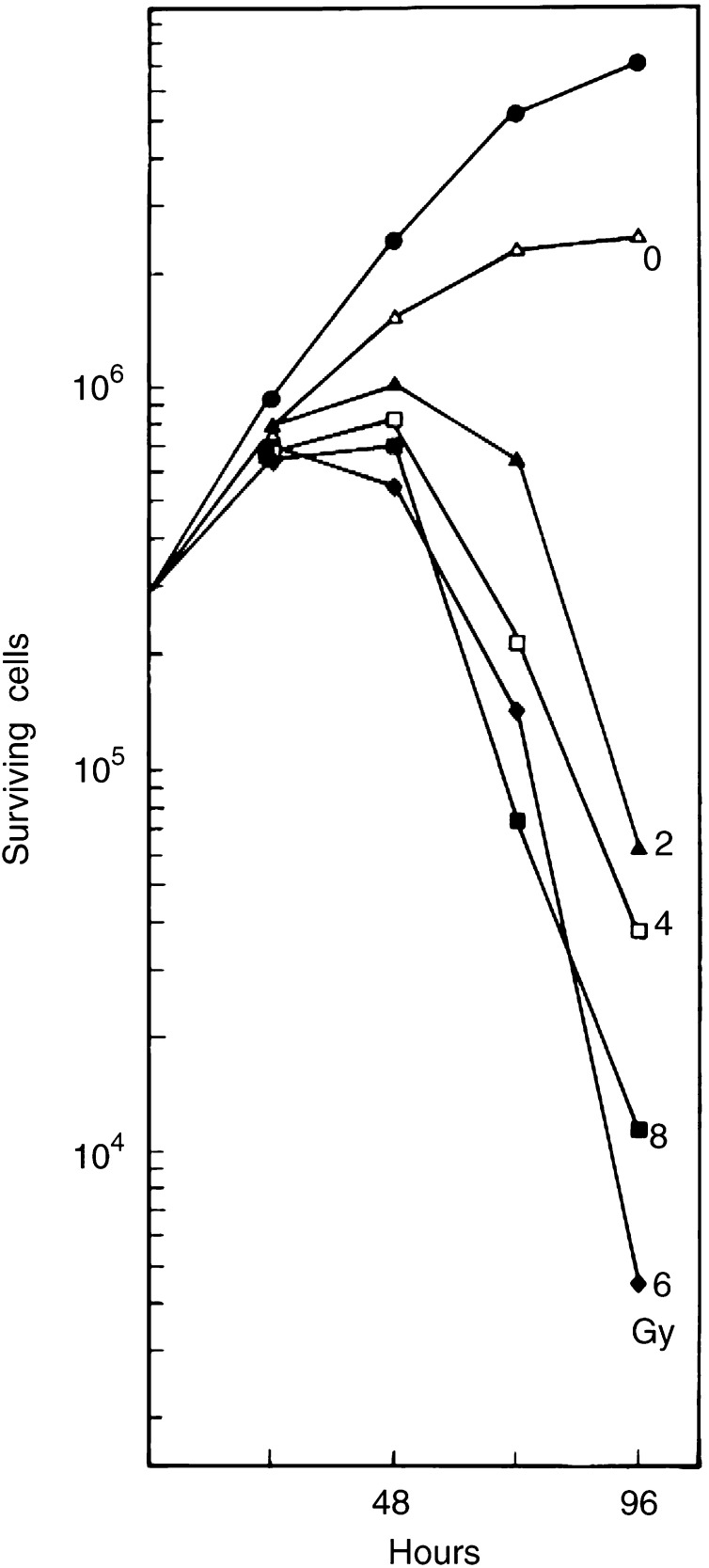
Effect of the radiation dose on the conditioning of the medium. Cultures of cells were irradiated with various doses of X-rays and 50 000 cells were incubated for 96 h. CMX was tested as described in Figure 1, with the difference that the cloning efficiency was used to calculate the surviving cell population. Cells were given fresh medium (closed circles) or the conditioned media from nonirradiated cells (open triangles), respectively, from cells that were irradiated with 2, 4, 6 or 8 Gy.

To demonstrate the time- and density-dependent expression of the cytotoxic property in culture medium with irradiated cells, CMX from 50 k and 200 k cells treated with 8 Gy X-rays was tested at 24 h intervals. Figure 3

shows the number of test cells after a 72 h exposure to CMX. A clonogenic assay was included. With both cell densities, the cytostatic potency of CMX increases after 48 h of conditioning. However, after 72 h of conditioning, 90% of the test cells in 50 k CMX are still clonogenic, whereas in 200 k CMX this is only 40% with fewer cells recovered. Figure 3 also shows the cell numbers in the 200 k donor cultures with irradiated cells during the 96 h conditioning period. Following an irradiation with 8 Gy, cell population growth is 2–3 times on average until after 72 h when the regression indicates the prevalence of cell death. The effect of 2–8 Gy X-rays on the medium cytotoxicity was detected on test cells with 50 k CMX after 96 h of conditioning (Figure 4

). As far as cell proliferation is concerned, there is no significant difference for CMX in the dose range of 2–8 Gy where all show approximately the same inhibition (as for 8 Gy in Figure 1a) with respect to 0 Gy or control medium (results not shown). Only after inclusion of the clonogenic data, the number of surviving cells shows an inverse relationship with the radiation dose and it appears that the cytotoxicity of CMX increases until 6 Gy.

These results demonstrate that irradiated H35 cells change the culture medium, which then further affects cell proliferation and survival. In addition to radiation-induced delayed cell death, medium conditioning appears to induce a different form of cell death, possibly apoptosis. This suggests that either one or more noxious compounds are released in the medium or that it becomes deprived of certain growth factors or some essential nutrients.

### Analysis of conditioned medium from irradiated cells and effect of cell density and radiation dose

**Figure 5:**
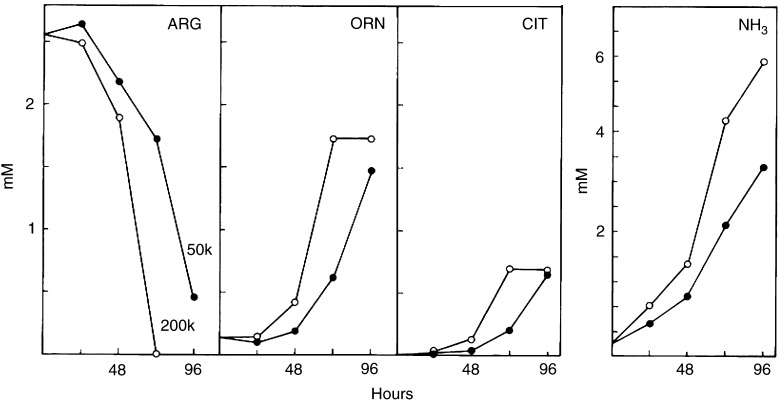
Composition of the ornithine cycle in the conditioned media from irradiated cells at different cell densities. Conditioned media from irradiated cultures were analysed for ornithine cycle compounds at various times following 8 Gy X-rays for 50 000 (50 k) and 200 000 (200 k) cells. Data are compatible with Figure 3.

**Figure 6:**
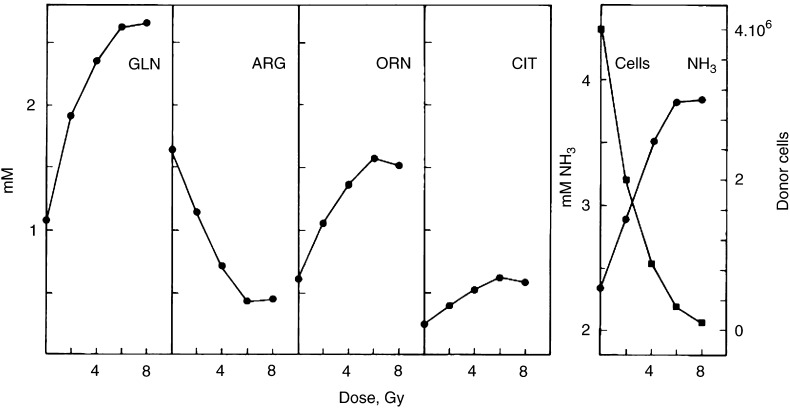
Composition of the ornithine cycle in the conditioned media from cells irradiated with various doses of X-rays. Conditioned media from cultures with 50 000 cells were analysed for ornithine cycle compounds at 96 h following irradiation with 0–8 Gy X-rays (see Figure 4). Cell numbers in donor cultures at 96 h are included. Glutamine is shown for comparison.

In an attempt to elucidate the nature and identity of the putative cytotoxic factor, CMX was analysed with available routines for medium compounds and various metabolic products. Interestingly, we found remarkable changes in the ornithine or urea cycle in response to increasing doses of X-rays. Whereas arginine rapidly disappears from the medium, ornithine, citrulline and ammonia are produced in large quantities. The effect of cell density on this shift in the ornithine cycle is shown in Figure 5

in CMX from 50 k or 200 k cells. The radiation-induced changes occur earlier when the cell density is higher. This corresponds with the toxicity data in the growth test (see Figure 3). The influence of the radiation dose is shown in Figure 6

. The changes in the ornithine cycle are detectable at 2 Gy and increase until 6 Gy with the same initial number of irradiated donor cells (50 k). This corresponds with the dose-dependent cytotoxicity of the conditioned media in the growth test (see Figure 4). The conservation of glutamine, which is indispensable for the energy supply of these cells, direction initial values of 2.5–3 mM with increasing radiation dose, probably reflects the inhibition of cell proliferation.

### Correlation of radiation-induced changes in the ornithine cycle with the cytotoxicity of the culture medium

**Figure 7:**
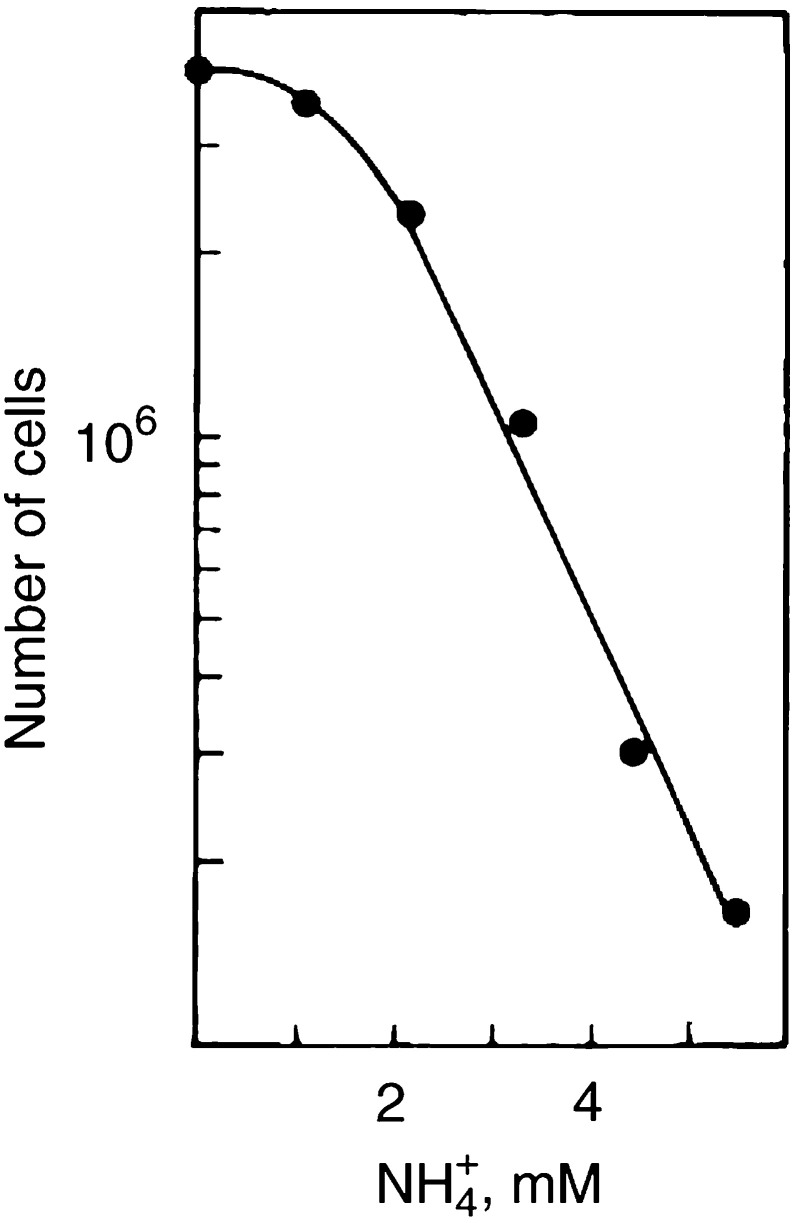
Effect of ammonium chloride on cell proliferation. Cells were incubated in fresh medium with various amounts of NH_4_Cl and counted after an incubation of 72 h. Except for the highest two concentrations with values of 0.6 and 0.5, respectively, the cloning efficiency was not affected.

**Figure 8:**
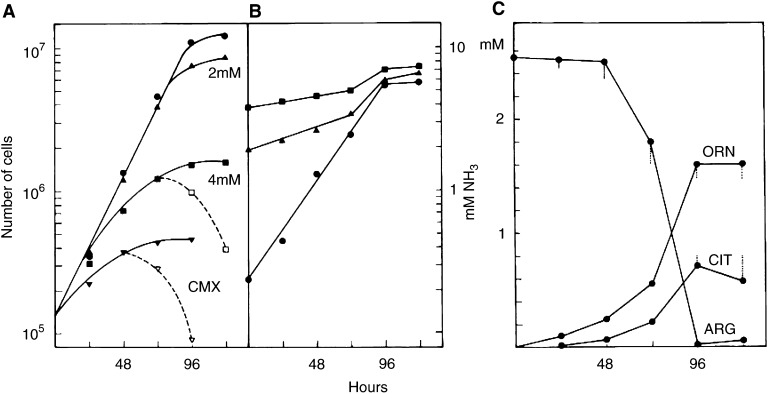
Comparison of the cytotoxicity of ammonium-supplemented media with conditioned medium from irradiated cells. Cells were grown in fresh culture medium provided with none (closed circles), respectively 2 and 4 mM NH_4_Cl or in CMX (8 Gy, 50 000 cells, 96 h). Dashed lines show the surviving cells with the cloning efficiency included (**A**). Parallel to cell countings, the concentrations of ammonia were determined in the culture media (**B**) as well as those of arginine, ornithine and citrulline (**C**). Vertical bars represent the maximum deviation in the ornithine cycle composition for the 2 and 4 mM NH_4_Cl media with respect to the control.

To investigate whether the changes in the ornithine cycle composition of CMX are related to the cytotoxic effect, a number of tests were done. The addition of 2–4 mM arginine to the arginine-depleted CMX does not abrogate its cytotoxicity. Neither ornithine nor citrulline is cytotoxic in normal cultures at concentrations of 1–2 mM (results not shown). However, the increase in ammonia appears to be critical. When H35 cells are exposed to increasing concentrations of ammonium chloride for 72 h, an increasing growth inhibition is noticed from about 2 mM NH_4_^+^ (Figure 7

). Cell proliferation is progressively inhibited and a correlation is suggested with the cytostatic effect of CMX (8 Gy/50 k/96 h), which contains 4 mM ammonia on average (see Figures 5 and 6). To test this, cell growth was compared in media with 0, 2 or 4 mM NH_4_Cl and in CMX. Figure 8

shows that 4 mM NH_4_Cl causes inhibition of cell proliferation within 72 h, whereas 2 mM has only a small effect relative to the untreated control (Figure 8a). This is approximately the amount of ammonia in normal growing cultures with 2.5 ×10^6^ cells. As compared to a medium with 4 mM NH_4_Cl, CMX is more cytotoxic and inhibition of cell proliferation already occurs within 48 h. The ammonia levels in the media (Figure 8b) indicate that cell proliferation runs out when the ammonia concentration rises above 2–2.5 mM (Figure 8a). Further analysis of the culture media (Figure 8c) reveals that arginine decreases after 48 h with concurrent increases of the ornithine and citrulline levels. Ammonium chloride has little influence on these changes.

Referrring to the previous section, it is understandable now that 50 000 cells after 2–8 Gy dose X-rays cause the accumulation of cytostatic amounts of ammonia, that is, 2.88–3.84 mM (see Figures 4 and 6). On the other hand, in 96 h cultures with 5000 (8 Gy) irradiated cells, the ammonia concentration is 1.5 mM on average, slightly above the 1.3 mM on average in the medium that is incubated for 96 h without cells. The increase is roughly one-tenth that produced by 50 000 cells, that is, approximately 2.5 mM.

### Other cytotoxic aspects of medium conditioned by irradiated cells 

**Figure 9:**
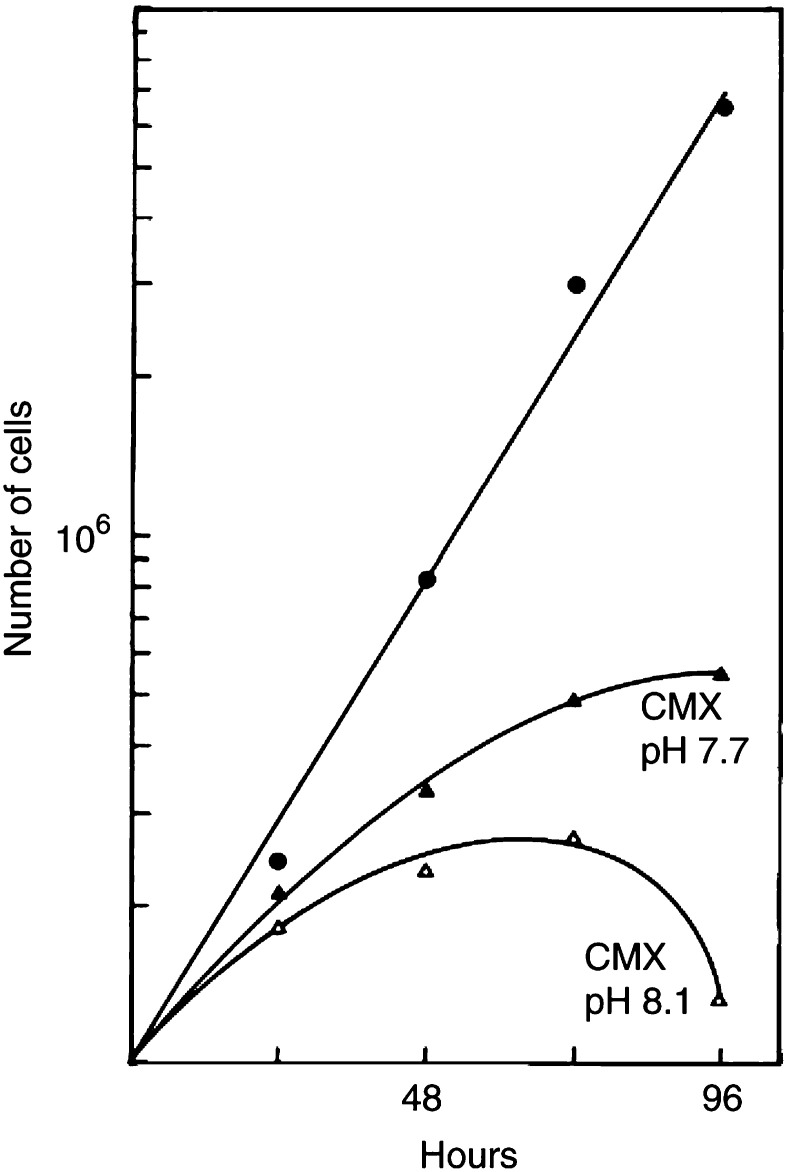
Influence of pH on cytotoxicity of CMX. The CMX pH of 8.1 was adjusted with HCl to that of control medium at 7.7. Conditioned media of 96 h were tested for the growth-promoting capacity as described in Figure 1. Ammonia concentrations in CMX (8 Gy 50 000 cells) and in the medium conditioned without cells (closed circles) were 4.6 and 1.5 mM, respectively.

The question was addressed as to why fresh culture medium with ammonium chloride is less cytotoxic than CMX with the same amount of ammonia. The observation that CMX is more alkaline than normal medium with or without NH_4_Cl suggests an explanation for the difference in cytotoxicity (addition of NH_4_Cl solution did not affect the medium pH). Ammonia in water reacts weakly alkaline as it binds the hydrogen ion. The pH of CMX was determined at 8.1 as compared to 7.7 in fresh medium. To investigate a possible pH effect of the CMX, it was adjusted to 7.7. As shown in Figure 9

, CMX at pH 7.7 is less cytotoxic than uncorrected CMX and its effect approaches that of NH_4_Cl (Figures 7 and 8). At 72 h about 80% more cells are present in CMX with the pH adjustment.

The depletion of arginine in CMX is not expected to affect its growth-promoting capacity to a significant extent as citrulline can fully replace arginine (see the next section). In conclusion, CMX cytotoxicity can be largely attributed to a toxic accumulation of ammonia in the medium through the irradiated cells.

### Intervention with the ornithine cycle and its influence on the conditioning process

After the demonstration that X-rays induce an excessive production of ammonia in the culture medium of these cells, which explains the cytotoxicity, we looked for conditions that could prevent it. Given the rapid breakdown of arginine in a medium of irradiated cells primarily into ornithine, we first investigated the effect of arginine-free culture medium. For the growth test then, arginine has to be added because the cells do not proliferate in an arginine-deficient medium. Clearly, cytotoxicity does not develop in the arginine-deficient CMX (Table 1

**Table 1:** Intervention in the ornithine cycle and the CMX-related cytotoxicity

**Modification to CMX**	**Arginine (mM)**	**Ornithine (mM)**	**Citrulline (mM)**	**NH_3_ (mM)**	**Number of test cells after 72 h**
None	1.05	1.17	0.50	3.85	0.46 (4.52)^a^
Norvaline	2.87	0.07	0.04	1.26	2.88 (3.12)^a^
L15-arg	0.006	0.20	0.06	1.55	4.64 (none)^a^
Citrulline	Nil	0.39	1.15	1.96	3.68 (4.79)^a^

a The number of test cells at 72 h (10^6^ cells) in the same media, but preincubated for 96 h without cells. Concentrations of citrulline (in arginine-deficient medium L15-arg) and of norvaline were 1 and 20 mM, respectively.

**Figure 10:**
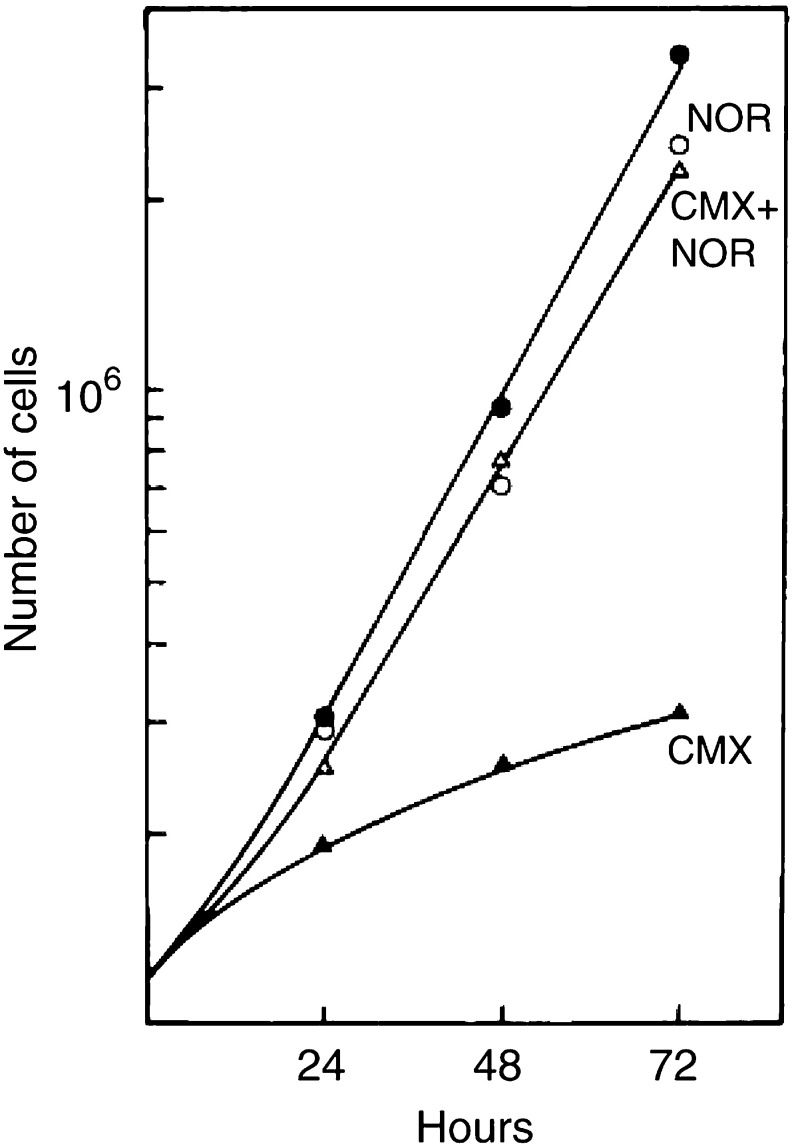
Effect of norvaline on cell proliferation in CMX. Cell proliferation was determined in CMX (8 Gy, 50 000 cells, 96 h) and in CMX with 20 mM norvaline (NOR) present during the conditioning period of 96 h and the growth test. Control media with or without norvaline were preincubated without cells. Ammonia in CMX changed from 4.35 at 0 h to 4.90 mM at 72 h. In CMX with norvaline these figures were 1.09 and 2.07 mM, respectively. In the control medium, ammonia changed from 1.12 to 2.05 mM at 72 h or from 0.93 to 1.69 mM with norvaline.

CMX of different compositions from 50 000 cells (8 Gy, 96 h) was tested for its growth-promoting capacity or analysed for ammonia and the ornithine cycle compounds.

). It was discovered that arginine can be replaced by citrulline without affecting normal cell proliferation. This also prevents expression of CMX toxicity and the surviving cells can even develop colonies in the same medium. The rapid decrease of arginine and its conversion primarily into ornithine indicates that the enzyme L-arginase, which catalyses this step, is important for the development of CMX toxicity. L-norvaline is a nontoxic inhibitor of arginase (Meijer *et al*, 1990), which effectively prevents arginine turnover into ornithine. In the presence of 20 mM norvaline, arginine is conserved, and ornithine and citrulline are hardly detectable in CMX (Table 1). Taking into consideration the slight inhibition of cell proliferation by norvaline, no significant cytostatic effect of CMX remains (Figure 10

). Similar to the replacement of arginine with citrulline, surviving irradiated cells can develop colonies in the presence of norvaline without the need of a medium change. The lack of toxicity in CMX, thus modified, is reflected by subtoxic ammonia levels; that is, below 2 mM (Table 1). The three protective modes also have in common that the ornithine levels are significantly reduced. Finally, it is remarkable that in the citrulline-based culture medium the citrulline concentration is hardly changed.

## DISCUSSION

Based on the present data, the expression of a cytotoxic ‘bystander’ effect in culture medium with irradiated hepatoma cells is largely explained by a toxic accumulation of ammonia. Although the precise mechanism is not clear, our data suggest that a different kind of cell death, possibly apoptosis, is induced in addition to the radiation-induced cell death. A similar, but significantly smaller, response is noticed with a medium from nonirradiated cultures, but only in the stationary growth phase with almost 100 times more cells involved. It thus appears to be a characteristic property of these cells, which is strongly enhanced after an exposure to X-rays and as such expressed at relatively low cell densitites. The effect of X-rays depends on the number of cells and the radiation dose. With a fixed number of irradiated cells, cytotoxicity is detectable after 2 Gy X-rays and increases until 6 Gy. This corresponds to increasingly cytotoxic ammonia concentrations.

The release of ammonia by irradiated cells is quite substantial. Following 8 Gy X-rays, cultures with only 50 000 cells can accumulate up to 4 mM ammonia within 4 days. In comparison to fresh medium that contains about 0.25 mM ammonia (see Figure 5), 1.1–1.5 mM is present in the medium after the same incubation period without cells. The formation of ammonia in the absence of cells is probably from a different origin as it is less affected by norvaline (see Figure 10 legend). In addition, the released ammonia probably causes the higher alkalinity in the culture medium, which further enhances the cytotoxicity.

The accumulation of ammonia correlates with the rapid turnover of arginine first into ornithine. Interruption of this process by the omission of arginine or the inhibition of L-arginase reduces or prevents the increases in ornithine and ammonia, and the related toxicity. This is a surprising observation, since the normal function of the ornithine cycle is to deposit ammonia into nontoxic urea. Clinical symptoms of hyperammonia are usually related to (heriditary) defects of the ornithine cycle and amelioration of the disease is sometimes affected by the suppletion of a deficient or restrictive component (Meijer *et al*, 1990). In the present study it is the absence of one such component, that is, arginine, which actually prevents the development of hyperammonia. This is in striking contrast to the known function of the ornithine cycle, as arginine appears to be the primary source for the excessive ammonia production here. Only in arginine-deficient citrulline-based medium, the ornithine cycle appears to be expressed in a more normal way, as the citrulline concentration hardly changes during an incubation with irradiated cells.

Arginine is indispensable for cell growth and survival (Scott *et al*, 2000). A possible explanation for the protection with citrulline is that the formation of arginine from citrulline is slow because of the rate-limiting enzymes argininosuccinate synthase and argininosuccinate lyase in comparison to its subsequent breakdown in ornithine and urea by arginase (Meijer *et al*, 1990). This probably supplies the cells with the necessary arginine without the adverse effects. Hepatic type-1 arginase is highly expressed in hepatocytes and has a high *V*_max_ (Wu and Morris, 1998). The present data suggest that its activity increases after an irradiation with X-rays. As far as we know, there is no information about possible regulatory effects of ionising radiation on arginase activity. The effectiveness of citrulline and its conservation suggest that the actual need of the irradiated nonproliferating cells for arginine is not as high as is suggested by its rapid breakdown. This indicates that the conversion of arginine into ornithine and ammonia follows a different pathway.

Although the primary source for the released ammonia appears to be clear, most of the pathway is still unsolved. The reversed formation of ammonia via the ornithine cycle is unlikely and undocumented. Investigation of this unique phenomenon is continuing and preliminary data indicate a further step towards the understanding of its mechanism. Results of a similar study with established cell lines from various human cancers show that the described phenomenon is not restricted to cultured cells of hepatocytic origin.
